# Mutation of a diacidic motif in SIV-PBj Nef impairs T-cell activation and enteropathic disease

**DOI:** 10.1186/1742-4690-8-14

**Published:** 2011-03-02

**Authors:** Ulrich Tschulena, Ralf Sanzenbacher, Michael D Mühlebach, André Berger, Jan Münch, Michael Schindler, Frank Kirchhoff, Roland Plesker, Cheick Coulibaly, Sylvia Panitz, Steffen Prüfer, Heide Muckenfuss, Matthias Hamdorf, Matthias Schweizer, Klaus Cichutek, Egbert Flory

**Affiliations:** 1 Division of Medical Biotechnology; Paul-Ehrlich-Institut; 2 Animal Facilities; Paul-Ehrlich-Institut; Paul-Ehrlich-Str. 51-59; 63225 Langen, Germany; 3 Institute of Virology, University of Ulm, 89081 Ulm, Germany; 4 Heinrich-Pette-Institut, 20251 Hamburg, Germany

## Abstract

**Background:**

The non-pathogenic course of SIV infection in its natural host is characterized by robust viral replication in the absence of chronic immune activation and T cell proliferation. In contrast, acutely lethal enteropathic SIVsmm strain PBj induces a strong immune activation and causes a severe acute and lethal disease in pig-tailed macaques after cross-species transmission. One important pathogenicity factor of the PBj virus is the PBj-Nef protein, which contains a conserved diacidic motif and, unusually, an immunoreceptor tyrosine-based activation motif (ITAM).

**Results:**

Mutation of the diacidic motif in the Nef protein of the SIVsmmPBj abolishes the acute phenotype of this virus. *In vitro*, wild-type and mutant PBj (PBj-Nef202/203GG) viruses replicated to similar levels in macaque PBMCs, but PBj-Nef202/203GG no longer triggers ERK mitogen-activated protein (MAP) kinase pathway including an alteration of a Nef-associated Raf-1/ERK-2 multiprotein signaling complex. Moreover, stimulation of IL-2 and down-modulation of CD4 and CD28 were impaired in the mutant virus. Pig-tailed macaques infected with PBj-Nef202/203GG did not show enteropathic complications and lethality as observed with wild-type PBj virus, despite efficient replication of both viruses *in vivo*. Furthermore, PBj-Nef202/203GG infected animals revealed reduced T-cell activation in periphery lymphoid organs and no detectable induction of IL-2 and IL-6.

**Conclusions:**

In sum, we report here that mutation of the diacidic motif in the PBj-Nef protein abolishes disease progression in pig-tailed macaques despite efficient replication. These data suggest that alterations in the ability of a lentivirus to promote T cell activation and proliferation can have a dramatic impact on its pathogenic potential.

## Background

Human and some simian immunodeficiency viruses (HIV, SIV) induce a slowly progressing immunodeficiency disease, preceded by an acute phase occurring within the first weeks of infection. The acute phase is often characterized by fever, rash, leukopenia, diarrhea, generalized lymphadenopathy, and anorexia associated with a peak of viremia and antigenemia [1-3]. In the early phase of infection, the gut-associated lymphoid tissue (GALT) rapidly becomes an active and preferred site of viral replication [4,5]. Primary viral replication in the GALT virtually eradicates memory CD4+ T cells in this compartment and is seen as a first strike of the virus against the immune system with long-lasting impacts [6-8]. While depletion of the GALT seems to be a common feature of lentiviral infections in primates [4-10], only in symptomatic courses of infection does the mucosal barrier become leaky resulting in translocation of microbial products and high levels of chronic immune activation [11,12]. In contrast, during asymptomatic infections the mucosal barrier recovers and the chronic phase is characterized by robust viral replication in the absence of immune activation [10,13]. However, which viral or host factors tip the balance between destruction or reconstitution of the mucosal barrier remains elusive.

The SIV macaque model provides a system to study lentivirus host cell interactions especially in the acute phase of infection and in the pathogenesis of acquired immunodeficiency syndrome (AIDS), mirroring especially the acute phase of HIV infections [5,14]. SIVsmmPBj (PBj), originally isolated from sooty mangabey monkeys (smm), induces a severe acute and lethal disease in pig-tailed macaques within 14 days of infection [15,16]. Characteristic acute symptoms are dehydration, severe lymphopenia, cutaneous rash and hemorrhagic diarrhea [17]. Pathological alterations observed during this phase include gastrointestinal villus blunting and fusion, mononuclear cell infiltration within the gastrointestinal tract, and high levels of virus replication in the GALT [18]. Similar pathological features, albeit in a milder form, are commonly observed in human AIDS patients, referred to as HIV enteropathy [4,19,20]. The severe acute pathogenicity of PBj is linked to the ability of the virus to induce activation and proliferation of infected resting peripheral blood mononuclear cells (PBMCs), which is associated with elevated levels of proinflammatory cytokines [21,22], such as IL-6 and TNF-α [23].

Multiple genetic elements have been described that influence the acutely lethal phenotype of PBj [24], and particularly the viral accessory protein Nef has been shown to play a critical role. An immunoreceptor tyrosine-based activation motif (ITAM) important for cell activation processes, located at the amino-terminus of Nef, has been described as one of the genetic determinants of SIV-PBj pathogenicity [25,26]. When reconstituted in the *nef *gene of the pathogenic SIVmac239, SIVsmmPBj-like features, as replication in resting PBMCs accompanied with lymphocyte activation [27,28] and induction of acute enteropathic pathogenesis [27-29] in inoculated macaques, were recovered with the respective mutated virus. However, while the reconstitution of the ITAM resulted in enhanced T cell activation and viral replication, it is still unclear if the high pathogenicity of this virus is mediated by its unusual ability to boost immune activation. Moreover, when the ITAM is transferred into an apathogenic lentivirus, its presence alone in Nef seems not to be sufficient for induction of acute pathogenicity [30,31].

The Nef protein is conserved in HIV and SIV and has been shown to be required for high viral loads and rapid progression to simian AIDS in infected rhesus macaques [32]. In addition, it has been suggested that loss of Nef´s ability to down-regulate CD3 and consequently block T-cell activation might be one reason for the high pathogenicity of HIV-1 in humans [33]. This hypothesis is supported by recent data showing that suppression of T- cell activation by Nef correlates with preserved T-cell counts in naturally infected sooty mangabeys [34]. Expression of Nef causes downregulation of a number of cell surface proteins, including CD4 [35], CD3 [36,37], and major histocompatibility complex (MHC) class I molecules [33,38]. Moreover, Nef modulates intracellular signaling pathways including the mitogen-activated protein kinase (MAPK) pathway via a conserved D-D-X-X-X-E motif present in the external loop region [39,40]. This evolutionary highly conserved signaling pathway, consisting of Raf-1, MEK1/2 (MAPK/ERK kinase) and the extracellular signal-regulated kinase (ERK) 1/2, is critical for cellular proliferation and activation processes [41]. These processes are involved in biological responses such as secretion of IL-2 [42,43], expression of cell activation markers such as CD69 and CD25 [44], activation of nuclear factor-κB (NF-κB) [45], up-regulation of lentiviral long terminal repeat (LTR)-dependent transcription [46] or other steps in the lentiviral life cycle [47,48].

We report here that mutation of the D-D-X-X-X-E motif in SIVsmmPBj-Nef (Nef202/203GG) leads to loss of MAPK-pathway activation without affecting the Nef protein's ability to stimulate viral replication in macaque PBMC. We exploited the unique phenotype of this mutant to study the impact of lentivirus induced T-cell activation and cellular proliferation. Pig-tailed macaques infected with PBj-Nef202/203GG virus exhibited viral loads similar to PBj-wt virus, while general immune activation was reduced. Most strikingly, PBj-Nef202/203GG virus infection did not show destruction of GALT and lethality as observed with PBj-wt virus. Altogether, the data presented here suggest a link between the ability of a lentivirus to induce T-cell activation and cellular proliferation with its ability to cause disease.

## Results

### Mutant PBj-Nef202/203GG virus shows similar replication kinetics and protein expression levels as wild-type PBj

**Figure 1:**
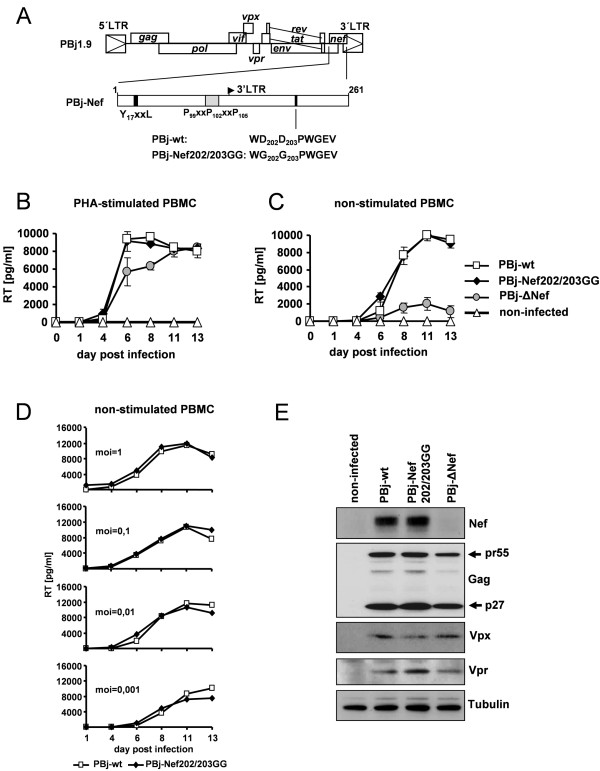
**Construction and replication kinetics of PBj-wt and PBj-Nef202/203GG**. (A) Schematic structure of SIV-PBj1.9 genome and PBj-Nef protein. The position of the ITAM (YxxL), SH3-binding motif (PxxPxxP), start of the 3' long terminal repeat (3' LTR) and the D-D-X-X-X-E motif are indicated. (B) or non-stimulated (C) primary macaque PBMCs from 6 animals were infected with the PBj-wt, PBj-Nef202/203GG or PBj-ΔNef virus with an MOI of 1. RT activity was measured in culture supernatants. Error bars, SD. (D) Analysis of RT activity upon infection of non-stimulated primary macaque PBMCs with serial dilutions of PBj-wt or PBj-Nef202/203GG virus. (E) Western blot detection of Nef protein expression in cell lysates of uninfected, PBj-wt-, PBj-Nef202/203GG- and PBj-ΔNef-virus infected C8166 T cells at day 8 p.i. Protein expression of viral Gag, Vpx, Vpr and cellular tubulin was analyzed as control.

To interfere with Nef-induced modulation of MAPK pathway, we introduced two nucleotide mutations into the *nef *gene of the infectious molecular virus clone SIVsmmPBj1.9, such that the two encoded consecutive aspartate residues (D) within the conserved D202-D203-X-X-X-E consensus motif in the C-terminal region of PBj-Nef were mutated into glycines (G). The resulting virus variant was termed PBj-Nef202/203GG (Figure 1A). The structural integrity of the mutant PBj virus particles was verified by electron microscopy (data not shown).

To examine the physiological consequences of this mutation, we first infected PHA-stimulated and non-stimulated PBMCs isolated from 6 different pig-tailed macaque donors *in vitro *with PBj-wt and PBj-Nef202/203GG using a multiplicity of infection (MOI) of 1. Infection with a virus variant which does not express Nef (PBj-ΔNef) was used as a control. Determination of reverse transcriptase (RT) activity in cell culture supernatants at different time-points after infection revealed indistinguishable replication kinetics of PBj-wt and PBj-Nef202/203GG in stimulated (Figure 1B) as well as in non-stimulated PBMCs (Figure 1C). In contrast, PBj-ΔNef replicated efficiently only in stimulated PBMCs (Figure 1B and 1C). Since effects of Nef on viral replication are more manifest at low MOI, RT activity was analyzed after infection of non-stimulated PBMCs using different MOI. In each case, similar replication kinetics of PBj-wt and PBj-Nef202/203GG were observed (Figure 1D). We next investigated whether the DD202/203GG mutation changed the expression level of Nef. Western blot analysis showed comparable Nef protein expression in C8166 T cells infected with PBj-wt or mutant PBj-Nef 202/203GG virus and, as expected, no detectable Nef protein in PBj-ΔNef infected cells. Comparable expression levels of viral Gag, Vpx and Vpr proteins as well as cellular tubulin were demonstrated (Figure 1E). Taken together, these results indicate that the introduced mutation does not affect the Nef protein expression level and the efficiency of SIVsmmPBj replication in activated and resting PBMC cultures.

### PBj-Nef202/203GG does not induce cell proliferation and activation of non-stimulated macaque PBMCs during replication

**Figure 2:**
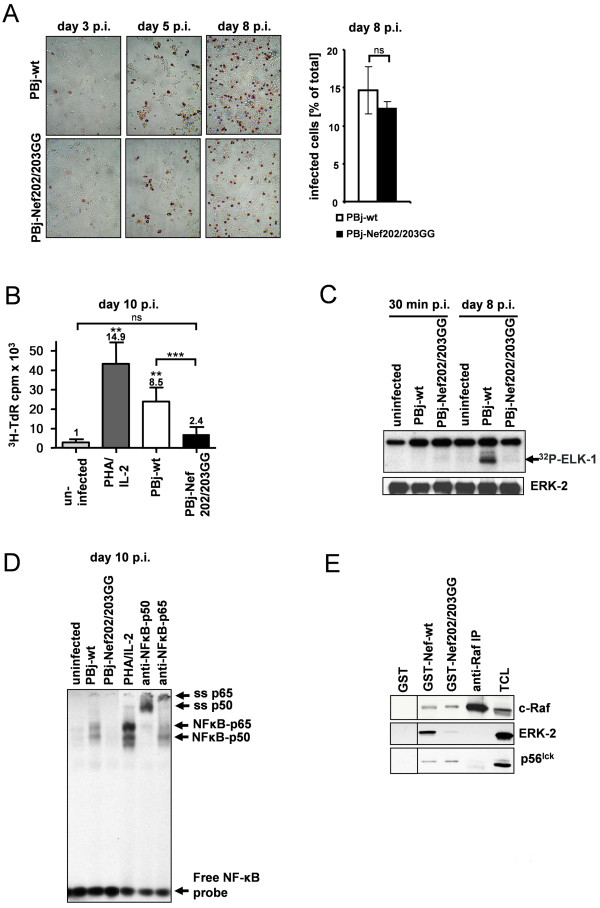
**Proliferation of PBMCs and activation of ERK1/2 and NF-κB upon infection with PBj-wt or PBj-Nef202/203GG virus**. (A) Analysis of virus gene expression by *in situ *immunostaining of PBj-wt or PBj-Nef202/203GG virus infected cell cultures with bar chart showing the percentage of infected cells at day 8 post infection as determined by cell counting. Magnification, 200 × (ns, *P *= 0.31). (B) Macaque PBMCs from 3 animals were infected with PBj-wt or PBj-Nef202/203GG virus. At day 10 p.i., cell proliferation was assessed by ^3^H-thymidine incorporation. PHA/IL-2 stimulated as well as non-stimulated uninfected PBMCs served as controls. Error bars, SD (**, *P *< 0.04 compared to control; ***, *P *= 0.036; ns, *P *= 0.21). Numbers represent stimulation index compared to non-stimulated uninfected cells. (C) *In vitro *ERK1/2 kinase activity. Non-stimulated macaque PBMCs were left untreated or infected with PBj-wt or PBj-Nef202/203GG virus. γ-^32^P-phosphorylation of ELK-1 quantified ERK1/2-activity. Western blot detection of ERK-2 served as loading control. (D) EMSA of NF-κB activation. Non-stimulated macaque PBMCs were left untreated, stimulated by PHA/IL-2 or infected with PBj-wt or PBj-Nef202/203GG virus. On day 10 p.i., NF-κB activity was assessed using a specific ^32^P-labelled oligonucleotide. The specificity of NF-κB binding complexes was confirmed by using NF-κB-p50 and NF-κB-p65 specific antibodies in supershift experiments. (E) Differential binding of PBj-wt and PBj-Nef202/203GG Nef to cellular signaling proteins. GST-PBj-Nef fusion proteins were used to precipitate potential binding partners from lysates of non-stimulated T cells. Precipitates were analyzed for Raf-1, ERK-2 and p56^lck ^by Western Blot. Precipitations with GST, Protein A-coupled anti-Raf-1, and total cell lysates (TCL) served as controls.

Previous studies revealed that SIVsmmPBj is able to replicate in non-stimulated, resting macaque PBMCs, concomitantly activating and inducing the proliferation of cells [16]. To analyze the replication and activation profile of the virus mutant, we infected primary non-stimulated PBMCs from 3 different macaque donors with PBj-wt or PBj-Nef202/203GG viruses (MOI of 1). As expected from the replication kinetics (Figure 1C), high numbers of infected cells were detected by SIV immunostaining in both cultures on day 5 and day 8 p.i. (Figure 2A). Quantification of the percentage of infected cells among total cell numbers in the respective culture on day 8 p.i. showed no significant difference between cultures infected with PBj-wt virus or the mutated PBj-Nef202/203GG virus, with a mean number of about 15% or 12% of total cell numbers infected, respectively (Figure 2A). Thus, no impairment of virus replication by the Nef-mutation could be observed, again. However, only PBj-wt virus, but not PBj-Nef202/203GG, consistently induced microscopically visible proliferation of PBMCs as detected by typical cell clusters and raise in cell numbers. Therefore, cell proliferation was measured by ^3^H-thymidine-incorporation on day 10 p.i. Consistent with previous results by Fultz et al. [16], infection of non-stimulated PBMCs with PBj-wt virus resulted in an 8.5-fold increase in thymidine uptake compared to uninfected non-stimulated PBMCs (Figure 2B), indicating the stimulation of cell proliferation by viral infection. In contrast, infection of non-stimulated cells with PBj-Nef202/203GG resulted only in a 2.4-fold enhanced ^3^H-thymidine uptake. A 14.9-fold increase in ^3^H-thymidin-incorporation was induced by control stimulation of non-infected PBMCs with phytohemagglutinin (PHA) and IL-2. These results indicate that PBj virus-induced PBMC proliferation is strongly impaired by the absence of the D-D-X-X-X-E motif in the Nef-protein.

Induction of cell proliferation requires mitogenic signaling via the ERK-dependent signaling cascade. Therefore, we analyzed the PBj virus-induced modulation of ERK1/2 kinase activity in non-stimulated primary macaque-derived PBMCs after infection with PBj-wt and mutant virus (MOI of 1) in an *in vitro *immuno-complex kinase assay. No activation of ERK1/2 was detected 30 minutes p.i. with either PBj virus, shown by the absence of phosphorylation of the ERK1/2 substrate ELK-1. However, a moderately increased ERK1/2 activity was observed on day 2 and 5 p.i. in PBj-wt infected cells (data not shown), and on day 8 p.i. a striking ERK1/2 activity was detected. In contrast, ERK1/2 activity was never observed in PBMCs infected with PBj-Nef202/203GG virus or in uninfected cells (Figure 2C). Thus, the D-D-X-X-X-E motif present in PBj-wt is essential for sustained activation of ERK in infected PBMCs.

As activation of the Raf-1-/MEK1/2-/ERK1/2 pathway is able to activate NF-κB, we analyzed the activity of this transcription factor in PBMCs 10 days p.i. in electrophoretic mobility shift assays (EMSA), monitoring binding of NF-κB p50/p65 extracted from infected cells to a ^32^P-labeled NF-κB specific probe. Infection of non-stimulated macaque PBMCs with PBj-wt virus (MOI of 1) induced enhanced binding of NF-κB p50/p65 heterodimeric complexes to the probe, demonstrating NF-κB activation (Figure 2D). This enhanced binding of NF-κB was comparable, albeit less pronounced to that observed in PHA/IL-2 stimulated cells. In contrast, infection with PBj-Nef202/203GG virus did not induce NF-κB activation. Specific binding of heterodimeric NF-κB-complexes was confirmed by adding an excess of unlabeled NF-κB specific probe as a competitor (data not shown) or by using NF-κB-p50 and NF-κB-p65 specific antibodies in supershift experiments (Figure 2D).

These results indicate that the D-D-X-X-X-E motif in SIVsmmPBj-Nef is critical for activation of Raf-1-/MEK1/2-/ERK1/2- and NF-κB- dependent signaling pathways. To test the physical interaction of Nef via its D-D-X-X-X-E motif with cellular Raf-1 *in vitro *as reported for HIV-1 [40], precipitation experiments were performed using recombinant GST-PBj-Nef proteins. Surprisingly, both recombinant Nef proteins precipitated Raf-1 (Figure 2E, upper). However, ERK-2 was only precipitated efficiently with GST-Nef-PBj-wt, suggesting that the D-D-X-X-X-E motif is required for recruitment of ERK-2 into the Nef-associated multiprotein signaling complex (Figure 2E, middle). Since the central proline-rich motif of HIV-Nef has been reported to be essential for connecting Nef to a number of signaling pathways, including interaction with the T cell specific kinase p56^lck ^[49], we analyzed functionality of both GST-Nef proteins by co-precipitation of p56^lck^. As expected, p56^lck ^co-precipitated with GST-Nef-PBj-wt and GST-PBj-Nef202/203GG in similar amounts (Figure 2E, lower).

### Nef202/203GG retains certain Nef functions, but reveals impaired capacity to downmodulate CD4, CD28, or CD3

**Figure 3:**
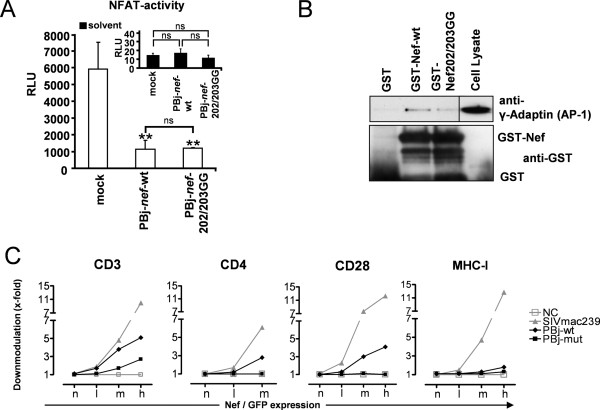
**Analysis of Nef functions**. (A) Analysis of NF-AT-activity. A.301 cells were co-transfected with a NF-AT-Luc-reporter plasmid and expression plasmids containing PBj-*nef*-wt, PBj-*nef*-202/20GG or pGL3-Basic as control. 16 h prior lysis, cells were stimulated with TPA and ionomycin (white bars) or solvent control (black bars). Mean of results of dual luciferase assays in triplicates is shown as relative luciferase units (RLU) (**, *P *< 0.04 compared to control; ns, *P *> 0.25). (B) Binding of the Golgi adaptor complex AP-1. GST-fusion proteins containing the C-terminal part (aa109-261) of PBj-wt and PBj-Nef202/203GG Nef was used to precipitate AP-1 from lysates of non-stimulated T cells, and GST served as control. Precipitates were analyzed by Western Blot using an AP-1 γ-subunit (γ-adaptin) detecting antibody (upper panel) or GST detecting antibody (lower panel). (C) Downmodulation of cell surface receptors. Analysis of Nef mediated downmodulation of CD3, CD4, CD28 and MHC-I was done and related to GFP reporter expression in Jurkat T cells transfected with respective pCG-nef-IRES-GFP plasmids as analyzed by FACS. For quantification, the levels of specific surface molecules´ expression (red fluorescence) were determined for cells expressing a specific range of GFP (n, no; l, low; m, medium; h, high expression). The extent of downmodulation (x-fold) was calculated by dividing the MFI obtained for cells transfected with the nef-minus plasmids by the corresponding values obtained for cells transfected with plasmids coexpressing Nef and GFP (NC, no Nef; SIVmac239, Nef of SIVmac239; PBj-wt, wt Nef of PBj; PBj-mut, Nef202/203GG of PBj). One representative out of 3 experiments displayed.

No structural implications for the folding of the Nef protein should be expected since these mutations are located in an external loop region of Nef (personal communication, B. Stauch, EMBL Heidelberg, Germany). Nevertheless, we confirmed typical Nef-associated properties and functions besides the preserved interaction with p56^lck ^(Figure 2E), which are not related to the ERK-mediated effects of the Nef202/203GG mutant. To analyze the functional activity of the mutated Nef protein, we first determined its ability to suppress NF-AT activation in A3.01 T cells [33,34]. We found that both the wt and the 202/203GG mutant Nef inhibited NF-AT induction by about a 5-fold downmodulation (Figure 3A), consistent with the published data for other SIV Nefs' [34]. GST-pulldown experiments further indicated structural integrity of the Nef202/203GG mutant protein by the association of γ-adaptin of the AP-1 adaptorprotein complex to both Nef-PBj-wt and Nef202/203GG (Figure 3B).

We next analyzed the surface expression of CD4, CD3, CD28 or MHC-I molecules on T cells transfected with pCG vector constructs expressing the wt-Nef, Nef202/203GG or, as positive control for downmodulation, Nef of SIVmac239, As expected, SIVmac239 Nef strongly downmodulated CD4, CD3, CD28 and MHC-I molecules. Interestingly, MHC-I was neither down regulated by wt or mutated PBj Nef (Figure 3C). As expected from previous studies [37,50], Nef202/203GG was attenuated in down-modulation of CD3 and defective in CD4 (Figure 3C), as well as CD28 down-modulation. The latter was expected, since the D-D-X-X-X-E motif in HIV-1 Nef has been recently described to be a novel AP-2 binding domain [51] and mediates contact of Nef to the V1H subunit of the vacuolar ATPase, which is most likely implicated in CD4 and CD28 down-modulation, as well [52,53].

These *in vitro *results show that Nef202/203GG enhances viral replication in the absence of mitogenic signaling and CD4 down-modulation and indicate structural integrity and function of Nef202/203GG.

### PBj-Nef202/203GG does not induce secretion of IL-2 in non-stimulated PBMCs

**Figure 4:**
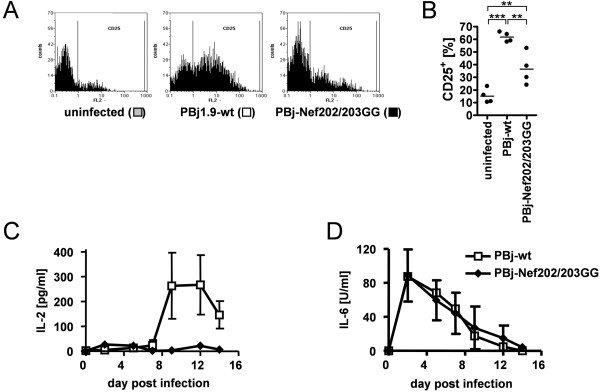
**Activation of infected macaque PBMCs *in vitro***. Non-stimulated macaque PBMCs were infected with PBj-wt or PBj-Nef202/203GG virus. (A and B) FACS analysis of cell surface expression of T cell activation marker CD25 on day 10 p.i. (A) Histograms of one representative animal. (B) Scattergram of CD25 surface expression on T cells of 4 different animals, horizontal bars represent means (**, *P *< 0.04; ***, *P *< 0.001). (C) IL-2 concentration and (D) IL-6 concentration was measured in tissue culture supernatants of infected PBMCs of 3 different animals by ELISA. Error bars, SD.

Cell proliferation and activation of ERK1/2 are important for induction of cellular responses such as the expression of cellular activation markers CD25 or secretion of IL-2. In infected non-stimulated PBMCs, flow cytometric analysis revealed that at day 10 p.i. a significantly higher proportion of CD25-positive cells was present in PBj-wt- as compared to PBj-Nef202/203GG virus infected PBMCs (62% vs. 38%, respectively) (Figure 4A and 4B). Furthermore, PBj-wt-infected non-stimulated PBMCs of 3 different donors on average secreted 267 pg/ml IL-2 as determined by ELISA, whereas PBj-Nef202/203GG-infected non-stimulated PBMCs did not secrete detectable amounts of IL-2 (Figure 4C). Remarkably, non-stimulated PBMCs infected with PBj-wt or PBj-Nef202/203GG both secreted comparable levels of IL-6 accumulating to approximately 90 U/ml at day 2 p.i. (Figure 4D).

Taken together, these data show that the D-D-X-X-X-E motif in PBj-Nef is required for induction of cell proliferation, activation of the mitogenic ERK1/2 signaling pathway and NF-κB, expression of cell surface activation markers CD25, and IL-2 secretion in infected PBMCs.

### Efficient replication of PBj-wt and PBj-Nef202/203GG *in vivo*

**Figure 5:**
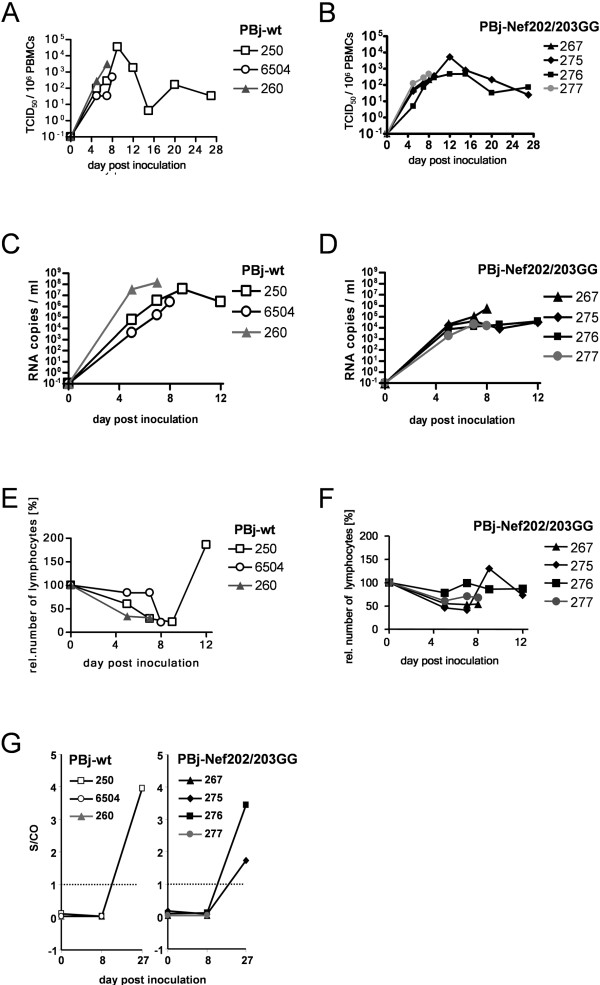
**Kinetics of plasma viremia and lymphopenia and seroconversion in macaques inoculated intravenously with PBj-wt or PBj-Nef202/203GG**. After inoculation of pig-tailed macaques with PBj-wt or PBj-Nef202/203GG virus blood samples were taken at different time-points p.i. and analyzed for cell associated viral load and relative lymphocyte counts. Data for animals #260 and #277, inoculated with the 10-fold virus dose, are shown in grey. (A and B) Cell associated viral load in the peripheral blood, determined by limiting dilution titration of PBMCs of infected macaques on C8166 cells, presented as TCID_50 _for animals inoculated with (A) PBj-wt virus or (B) PBj-Nef202/203GG. (C and D) Plasma viral load determined by quantitative RT-PCR on plasma of infected macaques, presented as genome copies / ml plasma for animals inoculated with (C) PBj-wt virus or (D) PBj-Nef202/203GG. (E and F) Total lymphocyte counts of infected macaques, related to preinoculation values. (G) Seroconversion of infected animals, as determined by crossreactive anti-HIV ELISA and shown as sample to cut-off value (S/CO). CO is indicated by dotted line.

After thorough analysis of mutated PBj virus *in vitro*, we aimed to analyze the effects of the Nef203/203GG mutation *in vivo*. Therefore, four pig-tailed macaques were infected intravenously (i.v.), three of them (animals #267, #275, #276) with 5 × 10^5^, and one (animal #277) with 5 × 10^6 ^infectious units (TCID_50_) of PBj-Nef202/203GG virus. In parallel, two macaques (#250, #6504) were infected with 6 × 10^5 ^and one (#260) with 6 × 10^6 ^infectious units (TCID_50_) of PBj-wt virus (Table 1). Blood samples of all animals were analyzed for cell-associated viral load, plasma viremia and lymphocyte numbers at different time-points p.i. We verified that the sequences encoding either the wild type or the mutated D-D-X-X-X-E motif were not mutated on day 9 p.i. from plasma-derived viral RNA from 10 sequenced independent isolated sequences (data not shown). Inoculated animals displayed a rapid rise in cell-associated viral load with maximal viral load at day 9 to 12 p.i. (Figure 5A and 5B) and PBj-wt- (Figure 5A) and PBj-Nef202/203GG-virus infected (Figure 5B) macaques showed comparable cell-associated viral load at all time points analyzed.

**Table 1:** Clinical symptoms observed after inoculation of macaques with different doses of PBj-wt or PBj-Nef202/203GG virus

Virus	Macaque	Dose (TCID_50_)	Anorexia	Dehydration	Haemor. Diarrhea	Apathy	Rash
**PBj-wt**	#260	5 × 10^6^	+	+	+	+	-
	#250	5 × 10^5^	+	+	+	+	+
	#6504	5 × 10^5^	+	+	+	+	-

**PBj-** **Nef 202/203GG**	#277	5 × 10^6^	-	-	-	-	-
	#267	5 × 10^5^	-	-	-	-	-
	#275	5 × 10^5^	-	-	-	-	-
	#276	5 × 10^5^	-	-	-	-	-

Occurrence of symptoms is indicated by "+", absence by "-".

Plasma viremia was determined by quantitative RT-PCR measuring viral genome copy numbers in the plasma. All animals inoculated with 5 × 10^5 ^TCID_50 _of either virus and animal #277, being inoculated with the 10-fold higher dose of PBj-Nef202/203GG virus, revealed similar plasma viral load around 10^4 ^RNA copies / ml on day 5 p.i. (Figure 5C and 5D). Animals inoculated with PBj-Nef202/203GG virus plateau on this level of plasma viremia showed mean titers of about 10^5 ^RNA copies / ml (Figure 5D), whereas macaques #250 and #6504 inoculated with PBj-wt virus displayed a further rise in plasma viral load titers up to 10^7 ^RNA copies / ml around day 8 p.i. (Figure 5C). Animal #260, inoculated with a 10-fold higher dose of PBj-wt virus, revealed increased replication kinetics achieving already on day 5 p.i. 10^7 ^RNA copies / ml plasma.

Following infection with PBj-wt virus, circulating numbers of lymphocytes dropped to an average of 25% of pre-inoculation values around day 8 p.i. (Figure 5E). In macaque #250, which survived the acute phase of disease, circulating lymphocyte numbers rebounded to above pre-inoculation values on day 12 p.i. Three of the four PBj-Nef202/203GG-infected macaques showed a decrease in the number of circulating lymphocytes to an average of 54% of pre-inoculation values and one animal, macaque #276, infected with the lower dose of PBj-Nef202/203GG virus, did not develop lymphopenia (Figure 5F). Replication of both PBj-wt virus and PBj-Nef202/203GG virus *in vivo *was followed by analysis of anti-SIV antibody induction. All animals tested had been seronegative up to day 8 p.i., as expected (Figure 5G). The animals surviving the acute phase of infection (#250, #275, and #276) revealed seroconversion by day 27 p.i. (Figure 5G), irrespective of the inoculated virus. Overall, these results indicate that mutation of the D-D-X-X-X-E motif does not abrogate the efficiency of virus replication.

### PBj-Nef202/203GG virus does not induce an acute lethal enteropathic disease in infected pig-tailed macaques

**Figure 6:**
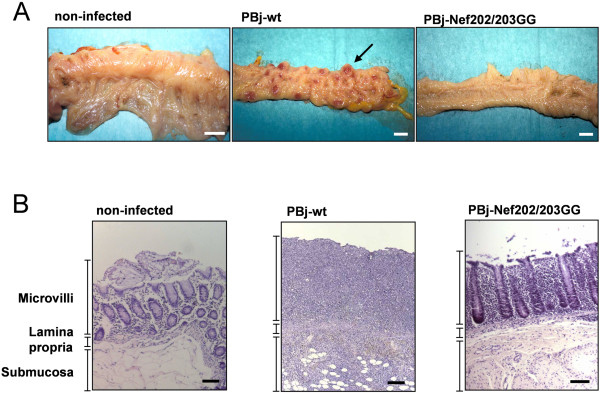
**Representative histopathology of infected macaques at day 8 p.i.**. (A) Macroscopic pictures of the colon of PBj-wt virus infected macaque #6504 and PBj-Nef202/203GG virus infected macaque #267. One characteristic ulcerative-necrotic lesion is indicated by an arrow. Scale bar, 1 cm. (B) Colon tissue sections stained with hematoxylin and eosin. PBj-wt virus infected macaque #6504 showed blunting and fusion of intestinal microvilli, resulting in complete loss of tissue structure, accompanied by massive infiltration of lymphocytes into the lamina propria. Macaque #267 showed intact microvilli architecture and moderate infiltration of lymphocytes. Scale bar, 100 μm.

All animals infected with PBj-wt virus developed a typical SIVsmmPBj-associated pathogenesis with characteristic fulminant disease symptoms including hemorrhagic diarrhea, anorexia, exicosis, apathy and rash, which were most severe between day 7 to 9 p.i. (Table 1). Macaque #260, which was infected with a higher dose of PBj-wt virus, developed massive acute disease symptoms at day 5 p.i. and succumbed to disease on day 7 p.i. PBj-wt virus infected macaque #6504 was euthanized on day 8 p.i., when showing comparable severe clinical symptoms. Subsequent complete histopathological analysis of spleen, liver, gut, and different lymph nodes revealed major pathological changes in the PBj-wt virus infected macaques #260 and #6540 as compared to a healthy animal. Such changes were most prominent in the gastrointestinal tract (Figure 6A), where blunting and fusion of intestinal villi, massive infiltration of lymphoid cells into the lamina propria (Figure 6B), and a massive hyperplasia of spleen and lymph nodes were observed.

In contrast to the animals infected with PBj-wt virus, none of the macaques infected with the mutant virus PBj-Nef202/203GG showed any of the clinical symptoms described above. Animals #277 and #267 were sacrificed on day 8 p.i. and showed a mild hyperplasia of spleen and lymph nodes, which was much less profound than in PBj-wt virus infected macaques. No macroscopical changes or lesions were found in the gastrointestinal tract (Figure 6A). Detailed histopathological analysis revealed minor fusions of intestinal villi and moderate numbers of lymphocytes in the lamina propria and the GALT (Figure 6B). Thus, these data indicate that the presence of the D-D-X-X-X-E motif in PBj-Nef is required for the induction of acute lethal pathogenicity in infected pig-tailed macaques.

### PBj-Nef202/203GG virus infected pig-tailed macaques showed reduced cytokine secretion and expression of activation markers on CD3^+ ^T cells

**Figure 7:**
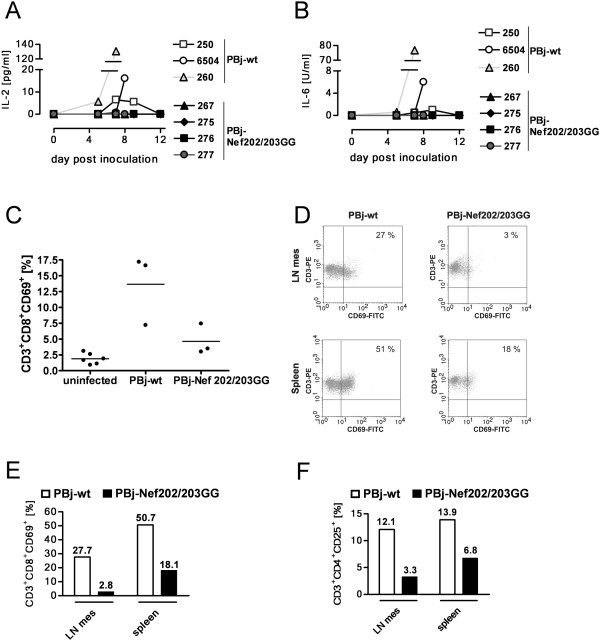
**Kinetics of plasma cytokine levels and activation markers on T cells of infected macaques *in vivo***. (A and B) Serum levels of (A) IL-2 and (B) IL-6 in blood samples taken at different time-points p.i., determined by monkey IL-2 and IL-6 ELISA, respectively. (C to F) Analysis of cellular activation markers on T cells at peak day of symptoms (day 8 p.i.) was determined by FACS. Percentage of positive cells is indicated. (C) Fraction of CD69 expressing CD3^+^CD8^+ ^T cells in the peripheral blood of PBj-wt or PBj-Nef202/203GG virus infected or uninfected macaques. Scattergram of individual animals, horizontal bars represents means. (D and E) CD69 surface expression on CD3^+ ^T cells from lymphatic organs (mesenterial lymphnodes, LN mes; spleen) of PBj-wt virus infected macaque #6504 and PBj-Nef202/203GG virus infected macaque #267. (D) Dot blot FACS analysis of representative individuals. (E) CD69 determined on CD3^+^CD8^+ ^gated lymphocytes. (F) CD25 on CD3^+^CD4^+ ^cells from LN mes and spleen of infected macaques.

*In vitro *studies described above suggested a role of the D-D-X-X-X-E motif in the release of cytokines. Therefore, the concentrations of IL-2 and IL-6 in the serum of inoculated animals were quantified by ELISA at different time-points p.i. All PBj-wt virus inoculated animals showed elevated IL-2 levels in the serum with a peak between day 7 and 9 p.i. (Figure 7A). The animals infected with the lower dose of PBj-wt virus revealed IL-2 serum levels of up to 16.1 pg/ml (macaque #6504) and 6.5 pg/ml (macaque #250). In the serum of macaque #260, infected with the higher dose of PBj-wt virus, 130.6 pg/ml IL-2 were measured at day 7 p.i. This indicates that the amount of IL-2 secretion might be dose-dependent. Animals inoculated with PBj-wt virus showed IL-6 serum levels of 1.0 (macaque #250), 6.0 (macaque #6504) and 76.8 U/ml (macaque #260), respectively (Figure 7B). In contrast, none of the PBj-Nef202/203GG virus infected macaques revealed detectable serum levels of IL-2 or IL-6 (Figure 7A and 7B).

As described, we observed different cell activation by PBj-wt and PBj-Nef202/203GG virus *in vitro*. Therefore, we determined the effect of the D-D-X-X-X-E motif on T cell activation *in vivo*. By FACS analysis, the expression of the early and late activation markers CD69 and CD25 was determined on T cells isolated from peripheral blood of infected animals. On the peak day of symptoms (day 7/8), an average of 14.7% of CD3^+^CD8^+ ^peripheral T cells expressed CD69 in PBj-wt virus inoculated macaques compared to 4.6% in PBj-Nef202/203GG virus infected macaques and 1.9% in non-infected macaques (Figure 7C).

Since the majority of activated T cells migrate to lymphatic organs, we analyzed activation of T cells in these tissues. In spleen and mesenterial lymph nodes (LN mes) of PBj-wt virus infected macaque #6504, a higher fraction of CD3^+^CD8^+^CD69^+ ^and CD3^+^CD4^+^CD25^+ ^T cells was found on day 8 p.i., as compared to PBj-Nef202/203GG virus infected macaque #267 (Figure 7D to F). The differences were most profound in the spleen represented by 51% CD3^+^CD8^+^CD69^+ ^spleenocytes in PBj-wt virus- compared to 18% in PBj-Nef202/203GG virus-infected animals, respectively. Thus, the ability of PBj-wt virus to stimulate T cells *in vivo *was diminished by mutation of the D-D-X-X-X-E motif within Nef, confirming the results obtained *in vitro*. Taken together, our data reveal that the D-D-X-X-X-E motif is important for both CD3^+ ^T cell activation as well as induction of IL-2 and IL-6 secretion *in vivo*. The activation status of T-cells in the GALT and the ability of the virus to induce secretion of these cytokines seem to be critical for the induction of enteropathy and the acute lethal SIVsmmPBj phenotype.

## Discussion

Ongoing and high levels of immune activation is regarded as a hallmark of pathogenic SIV and HIV infections during the chronic phase of infection [54,55]. To evaluate the impact of the ability of a lentivirus to cause T cell activation and cellular proliferation on the induction of disease, this study examined the pathophysiological consequence of two adjacent aspartate to glycine mutations within the conserved D202-D203-X-X-X-E motif in the C-terminal region of SIVsmmPBj-Nef. Infection of macaque PBMCs with PBj-wt virus induced activation of the Raf-MEK-ERK signaling pathway, activation of NF-κB, cell proliferation, expression of T cell surface activation markers and IL-2 secretion *in vitro*. The mutant virus PBj-Nef202/203GG failed to induce these physiological activities despite of displaying similar replication kinetics, the same level of Nef protein expression and conservation of inhibition of the induction of NF-AT activity as observed for the wild-type virus or protein. Moreover, the mutant virus lost its ability to down-modulate CD4 and CD28 and impaired the down-modulation of CD3 on infected T cells. These data indicate that the ability of SIVsmmPBj to replicate in non-stimulated, resting PBMCs is not dependent on the observed cellular responses associated with virus infection, which were lost in the PBj-Nef202/203GG virus.

As expected, infection of pig-tailed macaques with PBj-wt virus led to development of the characteristic acute enteropathic disease, accompanied by T cell activation as well as elevated IL-2 and IL-6 serum levels. In contrast, the PBj-Nef202/203GG virus neither induced acute enteropathic disease nor comparable T cell activation in infected animals, although efficient viral replication of the mutant was observed *in vivo*. In summary, the *in vivo *studies strongly indicate a selective role of the diacidic motif in T cell hyperactivation and enteropathic disease but not in virus replication.

Cell proliferation, activation of ERK1/2 and NF-κB, and secretion of IL-2 observed after PBj-wt virus infection *in vitro *seem to be mediated by interplay of Nef with the mitogenic signaling cascade involving the D-D-X-X-X-E motif. Hodge and coworkers demonstrated a direct interaction of Raf-1 and Nef of HIV-1 through this conserved motif [40]. In the present study, we confirmed the interaction of Raf-1 also with PBj-Nef, but in contrast to HIV-1-Nef, the interaction was not abolished by the two point mutations in the D-D-X-X-X-E motif. However, we detected a notable difference of wild-type PBj-Nef and mutant Nef protein in their capacity to recruit ERK-2 kinase into the Nef-associated signaling complex. Compared to wild-type PBj-Nef, a strong impairment of ERK-2 association with Nef202/203GG was observed that might be associated with the differential capacity to activate ERK. Most likely, the observed differences in IL-2 secretion, induction of CD69 surface expression, and NF-κB activation can be attributed to impaired ERK activation, as these cellular responses have been shown to be activated by the mitogenic signaling cascade [42,44,45,56]. However, we cannot conclude that the physiological effects of Nef are visible in infected cells, only. In contrast, a bystander effect might be also expected in uninfected cells due to the enhanced stimulatory cytokine secretion of infected cells and stimulation of uninfected cells, thereby. Moreover, other pathways might be also involved in the reported phenotypic differences between wild-type PBj-Nef and the mutant Nef protein, since the D-D-X-X-X-E motif has also been reported to be involved in interaction with AP-2 and V1H-ATPase [51-53,57]. Accordingly, our results confirm that mutation of the D-D-X-X-X-E motif is affecting the capacity of Nef to down-modulate especially CD4 [37,57] and CD28, using the AP-2 mediated pathway [58]. Interestingly, Nef202/203GG was still able to down-modulate CD3, albeit at lower efficiency (Figure 3D). The surface expression levels of CD4, CD3, CD28 or MHC-I molecules on T cells is affected by the endocytotic recycling pathway of cellular surface molecules. The regulation of these pathways is described to be associated to ERK-signaling [59]. Thus, a non-mutually exclusive additional effect of the introduced Nef mutations on the CD4, CD3, and CD28 surface expression levels mediated via the MAP kinase ERK signaling pathways is possible and might also alter pathogenesis *in vivo*.

The PBj-wt virus infection model displays exaggerated features in respect to mitogenic signaling and kinase activation most likely due to the presence of the ITAM motif in PBj-Nef, which may result in CD3/CD28 co-stimulus independency. The ITAM is a critical component of the CD3-induced T cell signaling pathway, known to activate cells via the Raf/MEK/ERK signaling cascade. It has already been demonstrated that mutations in the ITAM of PBj-Nef reduced acute pathogenicity [30]. Moreover, introduction of the ITAM into the Nef protein of the pathogenic SIV strain SIVmac239 by a single point mutation has resulted in a virus mutant displaying similar characteristics as SIVsmmPBj in vitro and *in vivo *[27-29]. Interestingly, an inactivation of the D-D-X-X-X-E motif in Nef of the already mentioned SIVmac239 leads to attenuation of pathogenicity and viral replication in macaques [50]. This observation has been linked to the loss of downmodulation of CD4 on infected cells by the respective virus mutant [50].

Most importantly, we exploited the unusual phenotype of the SIVsmmPBj model in triggering T cell activation to investigate the relative contribution of virally induced T cell activation on the pathogenic potential of a lentivirus. Although a reduction in RNA viral loads is observed in PBj-Nef202/203GG infected animals at 8 d.p.i., this may not exclusively be causative for the observed dramatic differences in pathogenicity. Cummulating evidence suggests that viral replication alone is not sufficient to cause disease. It has been demonstrated that general T cell activation is a better predictor of AIDS progression than viral loads [60,61]. Furthermore, absence of chronic immune activation, despite robust viral replication, is a common feature of asymptomatic natural infections with SIVsm and SIVagm [9,10]. Interestingly, experimental induction of immune activation in chronically SIVagm-infected African green monkeys has recently been reported to result in increased viral replication and CD4^+ ^T cell depletion [62]. On the other hand, re-inoculation of sooty mangabey monkeys with the pathogenic SIVmac239 strain that causes simian AIDS in rhesus macaques results in an asymptomatic course of infection [63].

It is conceivable that viral as well as host factors impact the course of infection and therefore the induction of disease. However, it is still unclear which determinants drive the chronic immune activation associated with disease progression. It has been proposed that microbial translocation caused by depletion of the GALT during the acute phase is a cause of systemic immune activation in progressive disease [11,12]. However, recent data show that depletion of the GALT is a common feature of symptomatic as well as asymptomatic courses of infections [10]. Remarkably, microbial translocation and destruction of the mucosal barrier did only occur in pathogenic lentiviral infections [10,11,64]. Thus, maintenance of the mucosal barrier is generally considered to be a host specific feature. Here, it is noteworthy that pig-tailed macaque already display a compromised gastrointestinal integrity on the microscopic level already in the absence of SIV infection, which is potentially explaining on the one hand the more rapid disease progression of SIV infected pig-tailed macaques to simian AIDS [65], and on the other hand the higher pathogenicity of SIVsmmPBj induced acute disease in pig-tailed macaques [66] as opposed to rhesus macaques.

The data presented herein demonstrate that subtle alterations affecting the ability of a lentivirus to cause T cell activation can have a dramatic impact on disease progression and the integrity of the mucosal barrier. Previously it has been suggested that HIV-1 is particularly pathogenic in humans because its Nef is unable to suppress CD3 and consequently T cell activation [33]. This hypothesis is supported by recent data showing that the ability of Nef to block T cell activation correlates with preserved CD4 counts in naturally infected sooty mangabeys [34]. The phenotype of the PBj-Nef202/203GG virus in pig-tailed macaques resembles the situation of asymptomatic SIV infections of sooty mangabeys or African green monkeys: robust viral replication and macroscopically largely intact mucosal barrier in the absence of chronic immune activation. This is even more remarkable in the light of the described compromised gastrointestinal integrity of pig-tailed macaques [65]. It is furthermore noteworthy that this phenotype was achieved by the sole alteration of two amino acids in Nef lowering mitogenic signaling in the infected cell.

Therefore, our results demonstrate that high levels of general immune activation and integrity of the mucosal barrier in response to a lentiviral infection are not exclusively inherent features of the host. Rather than this, subtle alterations in the ability of a lentivirus to cause T cell activation can have a dramatic impact on disease progression.

## Conclusions

The mutation of a conserved diacidic motif in the Nef protein of the SIVsmm strain PBj is sufficient to prevent acute lethal disease in pig-tailed macaques despite efficient replication *in vitro *and *in vivo*. This attenuated phenotype is paralleled by modified mitogenic signalling in infected PBMCs. These data reveal that an ITAM motif found in the Nef protein of this SIV strain has to work in tandem with the conserved diacidic motif of Nef in activation of immune cells and concomitant pathogenicity, suggesting a potential role of the latter motif for the pathogenic potential of immunodeficiency viruses. Thus, already minute changes affect the ability of a lentivirus to cause T cell activation and can have a dramatic impact on the respective viral pathogenic potential.

Moreover, the absence of high levels of immune activation *in vivo *in response to efficient infection by the mutant virus reveals that the extent of immune activation in infected animals in response to lentiviral infection is not exclusively linked to host species-specific factors, but also determined by virus-specific features. Thus, our data suggest that specific features of lentiviruses may have a profound impact on disease outcome of different species, allowing potential interference within the virus-host interplay in the establishment of pathogenic infections.

## Methods

### Cells

Primary macaque PBMCs were isolated by Histopaque Ficoll (Sigma, Taufkirchen, Germany) gradient centrifugation from peripheral blood of *M. nemestrina*. PBMCs used for subsequent *in vitro *assays and human C8166 cells (ECACC No. 88051601) were cultured in RPMI 1640 supplemented with 2 mM L-Glutamin, 10% FCS and antibiotics.

### Plasmids and virus

For generation of Nef-mutated SIVsmmPBj, plasmid pPBj1.9 encoding the infectious molecular clone SIV_smm_PBj1.9 [15] was digested with *EcoR*I/*Not*I. The resulting 2,432 bp fragment was subcloned into the plasmid pZeoSV2+. Site-directed mutagenesis of the *nef *gene was performed using the QuickChange Kit (Stratagene, La Jolla, USA) according to the manufacturer's protocol using forward (5'-ACAAACTTCTCAGTGGG**G**TG**G**CCCCTGGGGAGAGGTACTGGC-3') and reverse (5'-GCCAGTACCTCTCCCCAGGGG**C**CA**C**CCCACTGAGAAGTTTGT-3') primers carrying two central single nucleotides (bold) resulting in the mutation of the encoded aspartate residues 202/203 into glycines (underlined) of the *nef *gene. The mutated plasmid DNA was verified by sequencing. Subsequently, the mutated subfragment was cloned back into pPBj1.9 via *EcoR*I/*Not*I, yielding the plasmid pPBj1.9Nef202/203GG. To generate expression plasmids (pGEX6P-PBjNefwt / -PBjNefGG) for PBj-wt and GST PBj-Nef202/203GG Nef-glutathione S-transferase (GST) fusion proteins, respectively, the respective *nef *genes were cloned into the plasmid pGEX-6P2 (Pharmacia, Uppsala, Sweden) according to the manufacturer's instructions by PCR using forward primer (5'-CGGGATCCGGTGGCGTTACCTCCAAGAAG-3') and reverse primer (5'-CCGGAATTCTTAGCTTGTTTTCTTCTTGTCAGCC-3').

Wild-type or mutant virus was produced by transfecting pPBj1.9 or pPBj1.9Nef202/203GG plasmid DNA into human C8166 T cells using DMRIE-C (Invitrogen, Karlsruhe, Germany) according to the manufacturer's protocol. Supernatant was harvested 8 days after transfection and virus samples were stored at -80°C. The 50% tissue culture infectious dose (TCID_50_) was determined by limiting dilution infectivity titration into C8166 T cells.

### Animal experiments

Animal studies on pig-tailed macaques (*Macaca nemestrina*) were performed in accordance with the guidelines of §8 Abs.1 of the "Deutsches Tierschutzgesetz" (TierSchG, BGB1.1 S.1105). The animals were SIV-negative and free of concurrent infections. For infections, macaques were inoculated i.v. with 5 ml PBS containing either 5 × 10^5 ^or 5 × 10^6 ^TCID_50 _of PBj-wt or PBj-Nef202/203GG virus. Citrate-buffered anti-coagulated blood samples were collected on days 0, 5, 7, 9, 12 and 27 post inoculation as well as on the days the animals were sacrificed by i.v. injection of 5 - 10 ml of T61 (Intervet Deutschland GmbH, Unterschleissheim, Germany).

### Virus load, lymphocyte counts and tissue analysis

Cell associated virus load (TCID_50_) in the peripheral blood of infected macaques was determined by limiting dilution infectivity titration of PBMC into C8166 T cells. Plasma viremia was quantified by quantitative RT-PCR. For this purpose, viral RNA was isolated from plasma samples using the QIAamp viral RNA extraction Kit (Qiagen, Hilden, Germany) according to the manufacturer's instructions. Copy numbers of viral genomes were quantified utilizing the QuantiFast SYBR Green RT-PCR Kit (Qiagen) with the primer pair SIV_F02 (5´-GCAAATCCAGATGTGACCCT-3´) and SIV_R02 (5´-GGTGGGCCACAATTCATATC-3´) on a LightCycler Instrument (Roche Diagnostics, Mannheim, Germany) according to manufacturers´ instructions with an annealing/extension temperature of 62°C. Hematology, particularly determination of lymphocyte numbers, was performed with an automated hematology analyzer (Cell-Dyn 3500SL, Abbott Diagnostics, Santa Clara, USA). Complete pathohistological examination of sacrificed animals was performed using haematoxylin and eosin (H&E) staining according to standard protocols.

### ELISAs and RT activity test

For determination of interleukin (IL)-2 and IL-6 levels in cell culture supernatants and serum samples, monkey IL-2 and IL-6 ELISAs (Biosource, Nivelles, Belgium) were performed according to the manufacturer's protocol. To determine reverse transcriptase (RT) activity from cell culture supernatants, the Lenti RT Activity Kit (Cavidi, Uppsala, Sweden) was used according to the manufacturer's directions.

### Flow cytometry

FACS analysis was performed from EDTA anti-coagulated blood samples using the Immunoprep kit (Beckman Coulter, Fullerton, USA) according to the manufacturer's protocol. Samples were incubated for 30 min with fluorophor-conjugated α-CD3-FITC, α-CD4-PE, α-CD8-PerCP, α-CD69-PE, or α-CD25-PE monoclonal antibodies (BD Bioscience, Franklin Lake, USA) and analyzed with a FACScan cytometer (BD Bioscience). Only living cells were gated and analyzed.

### Proliferation assay

3 × 10^5 ^macaque PBMC were infected with an MOI of 1 and were labelled on day 10 p.i. with 1 μCi of [^3^H]-thymidine (GE Healthcare, Buckinghamshire, UK) for 18 h. [^3^H]-Thymidine incorporation was assessed using a Betaplate scintillation counter (Perkin-Elmer, Turku, Finland).

### In situ immunostaining

3 × 10^5 ^macaque PBMC were infected with an MOI of 1. After attaching cells to poly-L-lysin-coated plates (Sigma) and fixation with methanol at -20°C, infected cells were visualized by IPA-staining of viral proteins as described previously [67].

### Western blot analysis

SIVsmmPBj1.9 Nef was detected in lysates of uninfected, PBj-wt or PBj-Nef202/203GG virus-infected C8166 T cells or macaque PBMC by Western blot analysis using crossreacting anti-HIV-2 Nef rat monoclonal antibody Hom-HB5 as described previously [46,67]. Subsequently, the blot was reprobed using anti-SIV-Gag p27 (clone KK60, NIBSC, Hertfordshire, UK), anti-HIV-2-Vpx (clone 6D2.6, NIH AIDS Research and Reference Reagent Program, Rockville, USA), anti-SIV-Vpr (kindly provided by B. Hahn) and anti-tubulin (clone YL1/2, Abcam, Cambridge, UK).

### Kinase phosphorylation assay

For kinase assays, supernatants of macaque PBMC lysates were prepared and incubated with polyclonal rabbit anti-ERK1/2 antibodies (Santa Cruz Biotechnology, Santa Cruz, USA) followed by incubation with protein A agarose and precipitation, as described previously [68]. Precipitates were washed in lysis and kinase buffer as described [46], incubated in kinase buffer supplemented with 5 μCi of [γ-^32^P]ATP (GE Healthcare, Buckinghamshire, UK ) and 1 μg Elk-1 substrate (Cell Signaling Technology; Danvers, USA) for 15 min at 30°C. After termination of the reaction in sample buffer, samples were subjected to SDS-PAGE, electroblotted and analyzed by autoradiography.

### Electrophoretic mobility shift assay (EMSA)

EMSA was done as described [69] with modifications. In brief, equal amounts of uninfected, PBj-wt or PBj-Nef202/203GG virus-infected macaque PBMC were lysed by repeated freeze-thaw cycles on ice. For binding reactions, 3 - 5 μg samples of nuclear extracts were incubated at room temperature for 20 min in the presence or absence of unlabeled oligonucleotide or 1 μl of NF-κB-p50 and NF-κB-p65 specific antisera in a 20 μl reaction mixture as described [46].

### Luciferase assays for NF-AT-activities

For analysis of NF-AT activity, 0.5 μg of an NF-AT-luc reporter (Stratagene) was cotransfected with 0.5 μg Nef expression plasmids using a dual luciferase reporter system for normalization (Promega). Cells were grown for 32 h and stimulated with TPA (20 ng/ml) and ionomycin (5 μM) (both Calbiochem, Nottingham, UK) or incubated with solvent for 16 h.

### Assessment of downmodulation of CD3, CD4, CD28 and MHC-I by Nef

Analysis of Nef mediated downmodulation of CD3, CD4, CD28 and MHC-I was done as described elsewhere [34]. Briefly, pCG-vector constructs, carrying functional *nef *genes followed by an internal ribosome entry site (IRES) and the GFP gene were cloned and used to transfect Jurkat T cells using the DMRIE-C reagent as described [70,71]. CD4, CD3, MHC-I, CD28 cell surface expression and GFP reporter expression in Jurkat T cells transfected with the respective pCG-construct was analyzed by FACS. For quantification of Nef-mediated modulation of specific surface molecules, the levels of receptor expression (red fluorescence) were determined for cells expressing a specific range of GFP. The extent of downmodulation (x-fold) was calculated by dividing the MFI obtained for cells transfected with the nef-minus NL4-3 control by the corresponding values obtained for cells transfected with vectors coexpressing Nef and GFP.

### Precipitation with GST-PBj-Nef fusion proteins

GST-PBj-Nef fusion proteins were expressed in *E. coli *using plasmids pGEX6P-PBjNefwt / -PBjNef202/203GG and were purified according to manufacturer's instructions (Pharmacia). For precipitation, unstimulated T cells were lysed in Triton-X100 lysis buffer. Supernatants of lysates were incubated with 100 μg fusion protein or 2.5 μg anti-Raf-1 monoclonal antibody (BD Biosciences) for 4 h at 4°C. Precipitation and Western Blot analysis was performed as described [68] using anti-Raf-1, anti-ERK2 (Santa Cruz Biotech.), anti-p56lck (kindly provided by O. Janssen), and anti-γ-adaptin ($$) antibodies.

### Statistics

All *P*-Values were calculated using the two-tailed Student´s t-Test for heteroscedastic samples.

## Competing interests

The authors declare that they have no competing interests.

## Authors' contributions

UT, RS and MDM participated in cloning, molecular and biological characterization of recombinant viruses *in vitro*, participated in conduction and analyzed the *in vivo *experiments, and drafted the manuscript. AB characterized recombinant Nef protein. JM and MS participated in characterization of Nef functions *in vitro*. FK participated in design of the study. RP and CC participated in *in vivo *experiments. SPa participated in analysis of viruses. SPr participated in cloning of viruses. HM and MH contributed to *in vitro *analysis of viruses. MS and KC participated in the design of the study and in drafting the manuscript. EF conceived of the study, participated in its design and coordination and helped to draft the manuscript. All authors read and approved the final manuscript.
